# Evidence for the replication of a plant rhabdovirus in its arthropod mite vector

**DOI:** 10.1016/j.virusres.2024.199522

**Published:** 2025-01-04

**Authors:** Hideki Kondo, Miki Fujita, Paul Telengech, Kazuyuki Maruyam, Kiwamu Hyodo, Aline Daniele Tassi, Ronald Ochoa, Ida Bagus Andika, Nobuhiro Suzuki

**Affiliations:** a Institute of Plant Science and Resources (IPSR), Okayama University, Kurashiki 710-0046, Japan; b Tropical Research and Education Center, University of Florida, Homestead, FL 33031, USA; c Systematic Entomology Laboratory, USDA, MD 20705, USA; d College of Plant Protection, Northwest A&F University, Yangling 712100, China

**Keywords:** Rhabdovirus, Plant, Mite, Vector, Replication, mRNA, Small RNA

## Abstract

Transmission of plant viruses that replicate in the insect vector is known as persistent-propagative manner. However, it remains unclear whether such virus-vector relationships also occur between plant viruses and other biological vectors such as arthropod mites. In this study, we investigated the possible replication of orchid fleck virus (OFV), a segmented plant rhabdovirus, within its mite vector (*Brevipalpus californicus* s.l.) using quantitative RT-qPCR, western blotting and next-generation sequencing. Time-course RT-qPCR and western blot analyses showed an increasing OFV accumulation pattern in mites after virus acquisition. Since OFV genome expression requires the transcription of polyadenylated mRNAs, polyadenylated RNA fractions extracted from the viruliferous mite samples and OFV-infected plant leaves were used for RNA-seq analysis. In the mite and plant datasets, a large number of sequence reads were aligned to genomic regions of OFV RNA1 and RNA2 corresponding to transcribed viral gene mRNAs. This includes the short polyadenylated transcripts originating from the leader and trailer regions at the ends of the viral genome, which are believed to play a crucial role in viral transcription/replication. In contrast, a low number of reads were mapped to the non-transcribed regions (gene junctions). These results strongly suggested that OFV gene expression occurs both in mites and plants. Additionally, deep sequencing revealed the accumulation of OFV-derived small RNAs in mites, although their size profiles differ from those found in plants. Taken together, our results indicated that OFV replicates within a mite vector and is targeted by the RNA-silencing mechanism.

## Highlights

• RT-qPCR and western blot analyses showed an increasing OFV accumulation in mites.
• mRNA sequencing revealed the expression of OFV genes in both plants and mite vectors.
• Small RNA analysis confirmed viral-derived small RNA accumulates in plants and mites.
• Our analyses indicated that OFV can replicate within its mite vector.

## 1 Introduction

Many plant viruses are vectored by plant-feeding insects, with modes of transmission categorized as nonpersistent, semipersistent, persistent-circulative, and persistent-replicative (Whitfield et al., 2015). Several members of the plant RNA viruses, such as plant reoviruses, orthotospoviruses, tenuiviruses, and classical plant rhabdoviruses that cause agriculturally important crop diseases, could be transmitted in a persistent-replicative manner by vector insects such as aphids, thrips, and plant- and leafhoppers (Hogenhout et al., 2008). Besides insects, arthropod mites (Acari)—such as the flat mites (*Brevipalpus* spp.) and wormlike mites (eriophyoids)—possess representatives that could transmit several plant RNA viruses (de Lillo et al., 2021; Kitajima et al., 2003). Similar to insect-borne plant negative-stranded RNA ([-]RNA) viruses (orthotospoviruses, tenuiviruses, and classical plant rhabdoviruses), some other (-)RNA viruses (mainly dichorhaviruses) have been suggested to replicate in the mites, although no direct evidence of the viral replication has been reported (Kitajima and Alberti, 2014; Kondo et al., 2003). In the case of dichorhaviruses, although transmission ability retains at least 3 weeks, and potential virus factories (called viroplasms) and virions have been observed in the mite body (Kitajima and Alberti, 2014; Kondo et al., 2003), research on these viruses’ replication within mite vectors is limited. This is due to the challenges in handling and rearing these tiny vectors. As a result, the interactions and co-evolution of plant viruses with non-insect arthropod vectors remain largely unexplored (Whitfield et al., 2018).

*Brevipalpus* mites, belonging to the family Tenuipalpidae, are small arachnids, approximately 0.2–0.3 mm in length, that are known to transmit both dichorhaviruses (nuclear type; see below for their taxonomy and characteristics) and cileviruses (cytoplasmic type; positive-stranded RNA viruses with a potentially non-replicative mode of transmission) (Childers and Rodrigues, 2011; Freitas-Astua et al., 2018; Kitajima et al., 2010). These mite-transmitted viruses affect a variety of crops, including citrus, coffee, tea, and ornamentals, causing diseases that potentially lead to agricultural losses (Bastianel et al., 2010; Goodin and Figueira, 2019; Kondo et al., 2003; Ramos-González et al., 2023). Unlike many other plant viral diseases, both dichorhaviruses and cileviruses do not infect the natural hosts systemically, inducing only local lesion symptoms. These viral diseases are primarily found in Central and South America, where one of them, citrus leprosis, has long been a problem in citrus production. Citrus leprosis is primarily caused by cileviruses, but probably also by dichorhaviruses (Chabi-Jesus et al., 2023; Childers et al., 2003; Hartung et al., 2015; Ramos-González et al., 2023). Citrus leprosis is estimated to cause annual losses of up to US$60 million and acaricide costs of US$54 million for the vector control in Brazil's citrus belt, composed of the states of São Paulo and Minas Gerais (Bassanezi et al., 2019; Moreira et al., 2022). The orchid strain of the dichorhavirus (orchid fleck virus, OFV) was first described in Japan (Chang et al., 1976; Fuji et al., 2022). However, it has subsequently been reported in many regions worldwide and can cause systemic infections in several orchid species, including *Cymbidium, Calanthe* and *Dendrobium* spp. (Blanchfield et al., 2001; Kondo et al., 2003). Additionally, the OFV citrus strain has also been associated with citrus leprosis-like symptoms in citrus in Central America (Cruz-Jaramillo et al., 2014; Roy et al., 2020; 2015b). OFV is transmitted by *B. californicus* (Kondo et al., 2003), but other known dichorhaviruses are transmitted by or associated with other *Brevipalpus* species belong to the *B. phoenicis* sensu lato group, such as *B. yothersi, B. phoenicis* sensu stricto, *B. papayensis*, and *B. azores* (Chabi-Jesus et al., 2018; 2023; Nunes et al., 2018; Ramos-Gonzalez et al., 2017).

*Dichorhavirus* is a genus in the family *Rhabdoviridae* (order *Mononegavirales*) and was recently placed in the subfamily *Betarhabdovirinae* along with eight other genera of rhabdoviruses, including the two classical groups of plant rhabdoviruses, nucleorhabdoviruses and cytorhabdoviruses (Walker et al., 2022). Currently, six viruses are classified as members of the genus *Dichorhavirus* (Chabi-Jesus et al., 2018; 2023; Kondo et al., 2006; Ramalho et al., 2014; Ramos-Gonzalez et al., 2017; 2018). Dichorhaviruses have a bisegmented (-)RNA genome. RNA1 has five genes, encoding the nucleocapsid protein (N), phosphoprotein (P), putative movement protein (MP), matrix protein (M), and glycoprotein (G), and RNA2 encodes the large polymerase (L; RNA-dependent RNA polymerase, RdRP) (Dietzgen et al., 2018; 2014; Kondo et al., 2006). In the OFV orchid strain, these genes are expressed as polyadenylated mRNAs and are likely sequentially transcribed from RNA1 in the nucleus by the virally encoded L protein (Kondo et al., 2014). In addition, putative polyadenylated leader RNAs (non-coding RNAs), which are likely involved in the regulation of genome transcription/replication, are also transcribed from 3′-terminal leader and 5′-trailer regions (antigenomic strand) (Kondo et al., 2014).

In this study, we investigated the possibility of OFV replication within the mite vector using an OFV orchid strain maintained in Swiss chard plant (*Beta vulgaris* L. var. *cicla*) and a population of viruliferous *Brevipalpus* mites. We performed RT-qPCR, western blot and RNA-sequencing (RNA-seq) analyses of mRNA and deep sequencing of viral small RNA. Overall, the results of this study provide molecular evidence indicating OFV replication in mites.

## 2 Materials and methods

### 2.1 Mite strain carrying an OFV isolate

The OFV isolate Cym07 (orchid strain, subgroup I) was originally derived from a symptomatic *Cymbidium* sp. plant from Tokushima Prefecture in Japan (Kondo et al., 2017). A viruliferous *Brevipalpus* mite population (Cym07Bc_JP) was obtained from the same *Cymbidium* plant and maintained on *Beta vulgaris* var. *cicla* L (Swiss chard) seedlings, in a growth chamber or plant growth room (approximately 22∼24 °C, 16 h light/8 h dark).

### 2.2 Mite transmission test

Viruliferous *Brevipalpus* mites (Cym07Bc_JP) were used as an inoculation source to test for the viral transmission and symptom development on some orchid and non-orchid plants. Two orchid species, *Cymbidium* sp. and *Dendrobium* sp., were used for mite inoculation and the plants were maintained in a greenhouse without temperature control. Four non-orchid plant species—New Zealand spinach (*Tetragonia expansa* L.), quinoa (*Chenopodium quinoa* Willd.), *Nicotiana benthamiana* L. (a wild tobacco), and *Arabidopsis thaliana* L. (ecotype Col-0)—were used as non-orchid plant hosts for the inoculation test (Kondo et al., 1995). Non-orchid plants were grown in the plant growth room (approximately 22∼24 °C, 16 h light/8 h dark), while the orchids were maintained in a greenhouse without temperature control. Three plants were used for each orchid species and five plants for each non-orchid plant species. For mite inoculation, at least 10–20 or more mites per leaf were inoculated on the tested plants. For virus transmission test, at least 10–20 or more adult female mites per leaf were used for the tested plants.

### 2.3 Morphological study of mites

The *Brevipalpus* mite population (Cym07Bc_JP) maintained on Swiss chard or *Cymbidium* sp. was subjected to the morphological analysis (Kondo et al., 2017). Photomicrography of mite samples was performed using a binocular stereomicroscope with a built-in camera (Stemi 305 cam, Carl Zeiss, Oberkochen, Germany). Low-temperature scanning electron microscopy (LT-SEM) was performed with an S-4700 field emission scanning electron microscope (Hitachi High Technologies America, Pleasanton, CA, USA) after freezing mite samples in liquid nitrogen and coating them with platinum (Bauchan et al., 2019). LT-SEM studies were performed in the Electron & Confocal Microscopy Unit USDA-ARS by Dr. Gary R. Bauchan.

### 2.4 Isolation of total RNA from plant and mite samples, and RT-PCR and western blot analyses

For extraction of plant total RNA, OFV_Cym07-infected leaf samples of New Zealand spinach or Swiss chard with chlorotic spots (four to five weeks after mite inoculation) were used after careful removal of the infesting mites. To extract mite total RNA, viruliferous mites (Cym07 Bc_JP) samples were collected from OFV-infected Swiss chard plants (using leaves from at least 30–40 plants). After washing the leaves with distilled water containing approximately 0.05 % Triton X-100 to detach the mites (all stages) from the leaf surface, mites were collected using a low-speed centrifuge (Kubota model KN-70, Tokyo, Japan). Total RNA was then extracted using the Maxwell® RSC miRNA Tissue Kit for the Maxwell automated system (Promega, Madison, WI, USA) or the NucleoSpin RNA Plant and Fungi Kit (Macherey and Nagel, Düren, Germany) according to the manufacturers’ instructions. Total RNA concentration was estimated using a NanoDrop spectrophotometer (NanoDrop 2000c, Thermo Fisher Scientific, Inc., Waltham, MA, USA). For RT-PCR detection, first-strand cDNA synthesis was performed using Moloney-murine leukemia virus reverse transcriptase (Thermo Fisher Scientific Inc., Waltham, MA, USA) with random hexamers or oligo (dT)_15_ primer (Takara, Otsu, Japan). The cDNAs were then subjected to PCR amplification using Quick Taq HS DyeMix (Toyobo, Osaka, Japan). Three sets of primers were used for *N, P* and *L* genes and two sets of primers for reference genes (the glyceraldehyde 3-phosphate dehydrogenase, GAPDH and elongation factor 1α, EF1α) of *Brevipalpus* mite or Swiss chard (Table S1). The PCR conditions were as follows: 94 °C for 2 min; 30 or 35 cycles of 94 °C for 15 s, 55 or 53 °C for 30 s, and 68 °C for 1 min; and 68 °C for 10 min. Electrophoresis of the total RNA or PCR amplicon was performed on a 1 % (w/v) agarose gel in 1× Tris-acetate-EDTA buffer, and the gel was stained with ethidium bromide for visualization.

For the time-course RT-qPCR analysis, an OFV-free mite colony was first established by feeding on tea (*Camellia sinensis* (L.) O. Kuntze) leaves, a non-host plant for OFV (Kondo et al., 2003), for two months. The colony was then maintained on healthy Swiss chard plants. The absence of the virus was confirmed through the lack of symptoms in Swiss chard and by RT-PCR detection of both mites and infested plants (data not shown). For OFV acquisition, these mites were transferred to OFV-infected Swiss chard (detached leaves in a Petri dish at room temperature) and collected at 2 and 5 days post inoculation, respectively. The mites were sampled just before transferring to the infected plant and this time point was designated as 0 days post inoculation. Alternatively, after 12 h virus acquisition, some mites were transferred to healthy Swiss chard leaves and reared for a further 24 h to provide a sample for 36 h after virus acquisition. Total RNA from 30 adult mites per sample (0, 2 and 5 days or 0, 12 and 36 h after virus acquisition) was extracted using NucleoSpin® RNA Plus XS (Takara) and then cDNA was synthesized using the Quant Accuracy RT-RamDA cDNA Synthesis Kit (Toyobo). RT-qPCR was performed using the qTOWER3/G (Analytik Jena, Thuringia, Germany) with THUNDERBIRD SYBR qPCR Mix (Toyobo). The relative accumulation levels of virus RNAs were determined using the comparative Ct method (2^–ΔΔCt^ method) and normalized to putative *GAPDH* as an reference mite gene (Table S1).

Western blotting was basically performed as previously described (Das et al., 2022). The 40 adult mites per sample (0, 2 and 5 days after virus acquisition) were used for analysis. Total proteins were fractionated in 4–12 % SDS-PAGE gel (NuPAGE™ Bis-Tris Mini Protein Gels, Invitrogen, Waltham, Massachusetts, USA) and transferred to the polyvinylidene difluoride (PVDF) membrane. The blot membrane was sequentially incubated with 1000-fold diluted anti-OFV antiserum (Kondo et al., 2009) and 10,000-fold diluted Goat anti-rabbit IgG-HRP (horseradish peroxidase) conjugate (#7074, Cell Signaling Technology Inc., Beverly, MA, USA). For detection, the SuperSignal West Pico PLUS Chemiluminescent Substrate (Thermo Scientific) was used with FUSION chemical luminescence imaging system (SOLO.7S.EDGEM instruments Inc., Osaka, Japan).

### 2.5 RNA sequencing

rRNA depletion RNA sequencing and mRNA sequencing analyses were basically performed as previously described (Andika et al., 2019; Kondo et al., 2021). A total RNA fraction from local lesions of three OFV_Cym07-infected *T. expansa* leaves (75.2 ng/µL) was used for rRNA depletion-RNA sequencing. The cDNA library was constructed using the MGIEasy FS DNA Library Prep Set (MGI Tech, Shenzhen, China) after rRNA depletion with the QIAseq FastSelect –rRNA Plant Kit (QIAGEN, Hilden, Germany). For mRNA sequencing, polyadenylated RNA fractions were enriched from total RNA samples extracted from OFV_Cym07-infected *T. expansa* leaves (part of the above-mentioned sample) and viruliferous mites (Cym07Bc_JP) (∼50 mg with some distilled water) collected from OFV-infected leaves (112.0 ng/µL) using the KAPA mRNA Capture Kit (Kapa Biosystems, Wilmington, MA, USA), and then libraries were constructed using the MGIEasy RNA Directional Library Prep Set (MGI Tech). These libraries were then subjected to deep sequencing using the DNBSEQ-G400RS (MGI Tech, paired-end 150-bp reads). The cDNA library construction and sequencing were performed by Genome-Lead Co. (Kagawa, Japan). After quality checking and adapter trimming of the raw reads, the trimmed reads were de novo assembled using CLC Genomics Workbench version 20 (CLC Bio-Qiagen, Aarhus, Denmark) with default parameters (length fraction = 0.5; similarity fraction = 0.8). The assembled sequence contigs were then used as queries for BLAST analyses against the viral RefSeq dataset of the NCBI (the National Center for Biotechnology Information), resulting in the identification of nearly complete genomic sequences for OFV genomes. In parallel, sequence reads were mapped to the OFV genome or its gene regions using the “Map Reads to Reference” algorithm in the CLC Genomics Workbench with stringent mapping parameters (length fraction = 0.9; similarity fraction = 0.95).

### 2.6 Small RNA analysis

Small RNA-seq analysis was performed as described previously (Kondo et al., 2023). For small RNA sequencing, total RNAs from two OFV_Cym07-infected Swiss chard leaves (132.60 ng/µL and RIN 7.6) and viruliferous mites (Cym07Bc_JP) collected from OFV-infected leaves (49.2 ng/µL and RIN 8.4) were used for the cDNA library construction using the TruSeq Small RNA Library Prep Kit (Illumina, San Diego, CA, USA). Deep sequencing was then performed using the Illumina HiSeq 2500 (Illumina, 51-bp single-end reads). The cDNA library construction and sequencing were performed by Macrogen Japan Co. (Tokyo, Japan). Raw reads were first trimmed to remove adapters and filtered to retain reads within the size range of 15 to 32 nucleotides using the CLC Genomic Workbench. The resulting reads were then mapped onto the OFV_Cym07 genome with stringent mapping parameters (length fraction = 0.9; similarity fraction = 0.99) using the “Read Mapping” algorithm in the CLC Genomic Workbench. The virus-derived small RNA reads were subsequently analyzed in detail using the MISIS-2 program (Seguin et al., 2016).

## 3 Results

### 3.1 The mite strain Cym07Bc_JP transmits OFV to some orchid and non-orchid plants

**Fig. 1:**
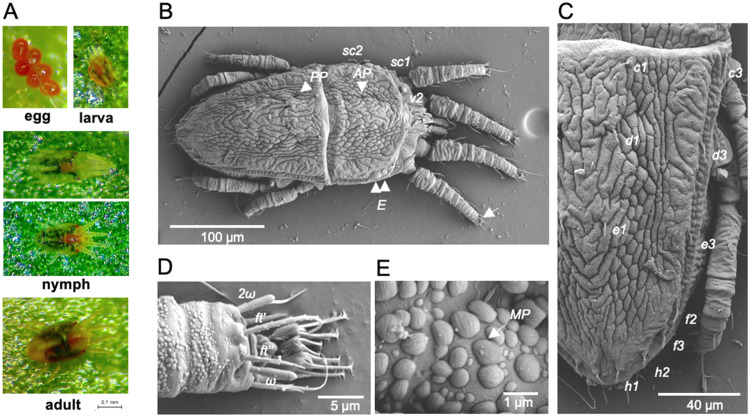
**Morphological characteristics of *Brevipalpus californicus* s.l. (strain Cym07Bc_JP). A**, Stereomicrographs of mites at different life stages. Upper left: an egg cluster; upper right: a larva; middle row (top and bottom): nymphs; lower row: an adult female. Scale bar represents 0.1 mm. **B–E**, low-temperature scanning electron microscopy of an adult mite sample collected from *Cymbidium* sp. The whole image (B) and a close-up of a part of the specimen (C–E). **C**, detail of the opisthosoma. **D**, distal part of the tarsus of leg II (white arrow in B). **E**, pattern of cuticular microplates. Eyes: *E*; prodorsal pore: *AP*; opisthosomal pore: *PP*; prodorsal setae: *v*_2_, *sc*_1_ and *sc*_2_; dorsal opisthosomal setae: *c*_1_, *c*_3_, *d*_1_, *d*_3_, *e*_1_, *e*_3_, *f*_2_, *f*_3_, *h*_1_, *h*_2_; festigials: ft and *ft’*; solenoid: *ω* and *2ω*; microplate *MP.* Other morphological information is shown in Fig. S2.

Swiss chard (*B. vulgaris* var. *cicla*) is a susceptible host of OFV and allows systemic virus infection (see below). Like on *Cymbidium* plants, the typical life cycle of the mite population (Cym07Bc_JP strain) could be observed on Swiss chard seedlings under stereomicroscope. All life stages including eggs, larva, protonymph, deutonymph, and adult (female), which display the typical reticulation patterns of *Brevipalpus* mites in dorsal view could be observed (Kondo et al., 2003) (Fig. 1A). The OFV_Cym07 isolate has been successfully maintained on this non-orchid host through mite transmission for >10 years since the virus was discovered (Kondo et al., 2017). Swiss chard is thus a suitable non-orchid plant for maintaining the OFV isolate and its viruliferous mite vector.

**Fig. 2:**
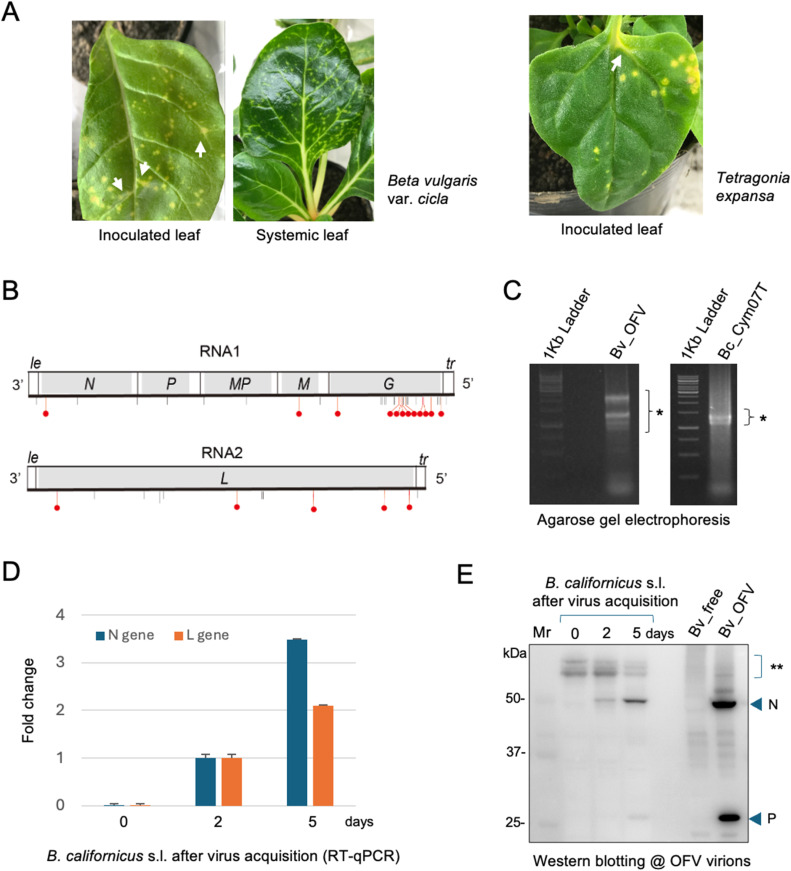
**Symptoms of OFV_Cym07 in plant hosts and detection of OFV_Cym07 in mites. A,** symptom in a OFV-infected plant of *B. vulgaris* var. *cicla* (left side) or *Tetragonia expansa* (right side) after infestation with viruliferous *B. californicus* s.l. (3 weeks after inoculation feeding for an inoculated leaf and 6 weeks for systemic leaves). **B,** the coding complete viral sequences were obtained from RNA-seq of the plant host (*T. expansa*) shown in the right panel in A. The ORF region is indicated by a light gray box. Sequences that differ from the first report are indicated by vertical lines at the bottom of each genomic RNA (see details of substitutions in Table S2). Substitutions with an amino acid sequence change are shown in red with a circle. *le*: leader and *tr*: trailer. **C,** Agarose gel electrophoresis of total RNA fractions from inoculated leaves (left panel: Bv_OFV) and infested mites grown on diseased Swiss chard plants (right panel: Bc_Cym07T). Both samples were subjected to a heat treatment at 68 °C for 10 min before loading. Asterisks indicate rRNA. **D and E,** Accumulation levels of OFV in the adult mites at different time points after virus acquisition (2 and 5 days) measured by RT-qPCR (*N* and *L* genes, D) or western blotting with rabbit antiserum against OFV virions (mainly N and P proteins, E). In the panel D, the data are presented as the fold change of viral accumulation (the value at 2 days was set to 1) with three technical replicates. In the panel E**,** double asterisks indicate the non-specific host signals that may be an indicator of a similar amount of mite samples loaded in each lane.

Inoculated orchids displayed, clear necrotic spots on the leaves of *Dendrobium* sp., while yellow spots (sometimes with necrotic rings) formed on the leaves of *Cymbidium* sp. approximately 6–8 months after infestation of the viruliferous mite strain Cym07Bc_JP (Fig. S1A and data not shown). Moreover, typical fleck symptoms (sometimes with necrosis) were observed on the new shoots of *Cymbidium* plants in the following spring (Fig. S1B), and no systemic symptoms were observed on newly emerged leaves of *Dendrobium*. OFV infection was confirmed by RT-PCR in symptomatic leaves of *Cymbidium* or *Dendrobium* plants, but not in asymptomatic newly emerged leaves of *Dendrobium* (data not shown). Non-orchid plants such as Swiss chard and *T. expansa*—but not quinoa, *N. benthamiana,* or *A. thaliana*—developed local yellow lesions 14–21 days after viruliferous mite inoculation (Fig. 2A and data not shown). In Swiss chard and *T. expansa*, the lesions often extended partially into the veins or petioles (Fig. 2A, arrows). After 4–6 weeks, systemic symptoms were observed in Swiss chard plants, usually on lower, developed leaves rather than newly emerged leaves (Fig. 2A). Notably, systemic infection rarely occurred on *T. expansa* and had fewer lesions when compared to Swiss chard (Figs. 2A; data not shown). Quinoa and *N. benthamiana,* which are susceptible to OFV through mechanical inoculation (Kondo et al., 1995; 2017), and *A. thaliana,* a known host of leprosis virus C (CiLV-C, a cilevirus) and many dichorhaviruses (Arena et al., 2017), were not infected with OFV through the mite inoculation. This may be an interesting feature to note, because these plants are unsuitable for mite infestation, and because *A. thaliana* is a non-susceptible host for OFV at least its orchid strain.

Sequencing of the OFV_Cym07 genome was done through RNA-seq analysis using total RNA extracted from the local lesion areas on *T. expansa* leaves. The results revealed several amino acid substitutions in the RNA1 and RNA2 coding proteins, particularly the G protein, as compared to the original sequence of OFV_Cym07 deposited in the GenBank database (Fig. 2B and Table S2).

### 3.2 Morphological characteristics of the mite strain Cym07Bc_JP

DNA sequencing of the mitochondrial *COI* (cytochrome c oxidase subunit I) gene of the mite population Cym07Bc_JP (accession no. LC222647) revealed that the sequence is closely related to that of *Brevipalpus californicus* Banks (Kondo et al., 2017). Further morphological examination of acknowledged *Brevipalpus* characters was performed using LT-SEM based on the general morphological features of *Brevipalpus* mites (Avalos-Cerdas et al., 2023; Welbourn et al., 2003). LT-SEM observation was performed by Dr. Bauchan (Electron & Confocal Microscopy Unit USDA-ARS) using *Brevipalpus* specimens obtained from mites that had infested *Cymbidium* and Swiss chard plants (Figs. 1B–E and S2). Our mite specimens showed the following morphological characteristics:(i) prodorsum with folds of irregular reticles (Fig. S2A); (2) opisthosoma with seven pairs of dorsolateral setae, setae *f2* present, cuticle between *c1-c1* and *e1-e1* similar in reticulation as central area of prodorsum; cuticle posterior *e1-e1* with V-shaped folds; sublateral furrow present, with well-defined cells, rectangular to square in shape bordering along the furrow (Fig. 1B and C); (iii) tarsus of leg II with two rod-shaped solenidia (*omega*) (Fig. 1D); (iv) separate, individual, round-to-ovoid cuticular microplates with fine longitudinal ridges on the surface (Fig. 1E); (v) palps four-segmented (Fig. S2B); (vi) ventral cuticle between legs III and IV was mostly longitudinal long cells; between *4a-4a* and ventral plate reticulate to verrucose with small and mid-cells (Fig. S2C); and (vii) ventral plate reticulated with cells broader than longer, organized in transverse bands formed by fusioned cells; genital plate with uniform narrow transverse bands and folds (Fig. S2D). Based on this morphoanatomical evaluation, particularly with the presence of two solenidia on tarsus II and the opisthosomal setal pair *f2*, the mite strain Cym07Bc_JP possesses the typical characteristics of the *B. californicus* species group (Avalos-Cerdas et al., 2023; Beard et al., 2012). Thus, it appears that this mite strain belongs to one of the sensu lato species of the *B. californicus* complex.

### 3.3 RT-PCR and western blotting analyses of OFV in plants and in viruliferous mites

Total RNA was extracted from viruliferous mite samples, which were collected from OFV-infected leaves using the detergent-wash method (see Materials and Methods). Agarose gel electrophoresis of the total RNA fractions extracted from the mite samples showed two close bands with a size similar to that of 18S rRNAs (Fig. 2C). This differed from the common pattern of plant total RNA, which shows two major bands corresponding to 28S and 18S rRNAs. In the case of insects and also in other arthropods (including mites), at higher temperatures (around 40–60 °C), 28S rRNA appears to be cleaved into two fragments, α and β, yielding two fragments with a size similar to that of 18S rRNA (McCarthy et al., 2015; Winnebeck et al., 2010). OFV was detected in the viruliferous *B. californicus* s.l. (Cym07Bc_JP) mite samples using primers specific for the *N, P*, and *L* genes, although amplification products obtained from mite samples were markedly lesser than from OFV_Cym07-infected Swiss chard plants (Fig. S3A). Note that plant mRNAs (*BvGAPDH* and *BvEF1*α) were not detected in the mite samples (Fig. S3B).

Using OFV-free and OFV-exposed mite colonies, OFV accumulation in mites at different time points after virus acquisition was examined by RT-qPCR and western blotting. In RT-qPCR analysis, OFV was detected at 2 days post virus acquisition but not at 0 days after virus acquisition (virus-free mites), while at 5 days post virus acquisition, virus accumulation was approximately 3.5- (*N* gene/RNA1) and 2.1 (*L* gene/RNA2) fold higher than that at 2 days post virus acquisition (Fig. 2D). Plant mRNA (*BvGAPDH*) was not detected in these mite samples (data not shown). In western blot analysis, the detection signals for structural proteins of OFV (especially for N protein) also increased significantly 5 days after acquisition in mites (Fig. 2E). In another experiment when the mites were transferred to healthy plants after 12 h virus acquisition and reared for an additional 24 h, RT-qPCR analysis showed a similar increasing pattern of virus accumulation after virus acquisition by the mites (Fig. S3C). These results strongly suggested that OFV replicates in mite (*B. californicus* s.l.) vectors. It was also confirmed that the mites showed the virus transmission ability to the healthy plants after 5 days virus acquisition (Fig. S3D).

### 3.4 RNA-seq analysis of OFV in plants and in viruliferous mites

Because the genome expression of OFV requires the transcription of polyadenylated mRNA (poly[A]^+^ RNA) in the host plant (Kondo et al., 2014), we carried out RT-PCR targeting mRNA using the oligo-dT primer for cDNA synthesis to investigate the possibility of viral gene expression in the mite vector. The *N, P*, and *L* genes were amplified from the mite sample, although the amplification levels were lower than those obtained using random primers for cDNA synthesis, which likely amplified both genomic RNAs and mRNAs (Fig. S3E). These results suggest that OFV mRNAs (at least for *N, P*, and *L* genes) are also expressed in mites.

**Fig. 3:**
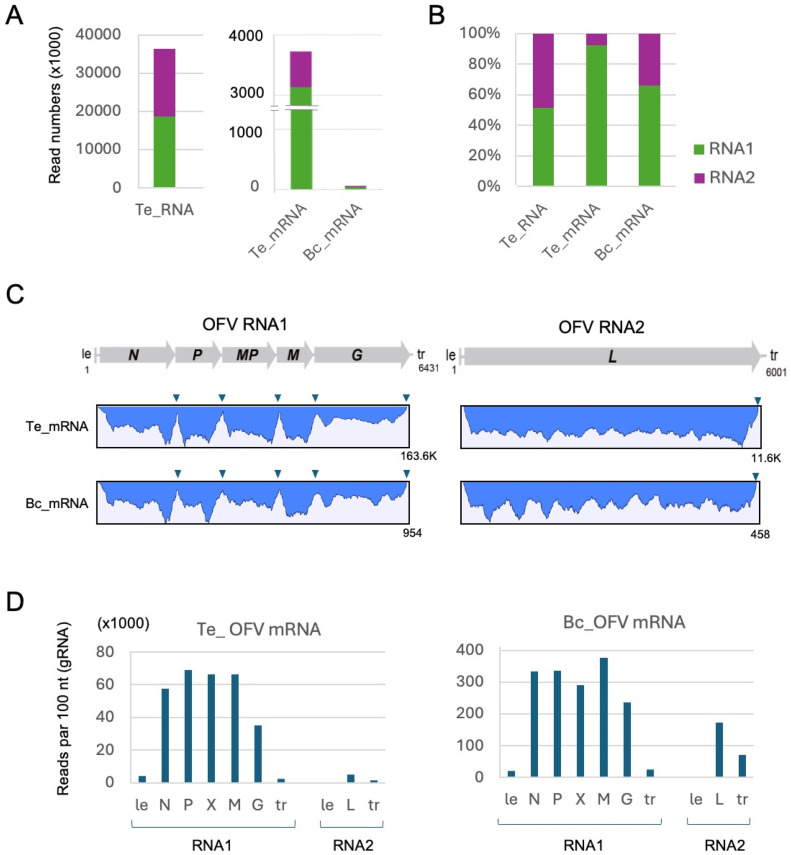
**Analysis of OFV_Cym07 gene expression using RNA-seq. A**, number of reads mapped to the OFV_Cym07 reference sequence (**A**). **B,** proportion of reads mapped to RNA1 and RNA2 segments (**B**). RNA-seq reads obtained from mRNA libraries of host plant (OFV-infected *T. expansa* leaves, Te_mRNA) and viruliferous *B. californicus* s.l. (Bc_mRNA) samples were used for mapping. RNA-seq data of *T. expansa* total RNA (rRNA-depleted sample, Te_RNA) were included for comparison. **C,** read depth coverage of the entire OFV_Cym07 genome sequences derived from the RNA-seq analyses of *T. expansa* leaves and *B. californicus* s.l. samples. **D,** viral reads from *T. expansa* leaves and *B. californicus* s.l. samples mapped to each gene of the OFV segment (vertical axis shows number of reads per 100 bases).

To further investigate the genome expression of OFV in mites, RNA-seq analysis was performed using the poly(A)^+^ RNA enriched fraction (from ∼50 mg of total RNAs) obtained from viruliferous *B. californicus* s.l. samples (Cym07Bc_JP). RNA-seq generated a total of 47.4 million (M) and 48.3 M raw reads from OFV_Cym07-infected *T. expansa* leaves (Fig. 2A) and viruliferous *B. californicus* s.l. (Cym07Bc_JP) samples, respectively. Bioinformatic analyses revealed that 3.9 M reads (4.11 %) from *T. expansa* and 0.03 M reads (0.03 %) from the mite samples were mapped to the OFV_Cym07 genome sequence (Figs. 3A and S4). Of these mapped reads, 92.3 % and 7.7 % of reads from the leaf dataset were aligned to the OFV RNA1 and RNA2, respectively, while 65.8 % and 34.2 % of reads from the mite dataset were aligned to the OFV RNA1 and RNA2, respectively (Fig. 3B). Notably, in both data sets, a significantly lower number of reads were mapped to the non-transcribed regions (gene junctions, marked with an arrowhead) between genes in RNA1 (Fig. 3C, Tx_mRNA). Additionally, in both datasets, reads were also mapped to the leader ([+] leader RNA) and trailer ([-] leader RNA, antigenomic strand) regions located at the end of the OFV genome, which transcribe short polyadenylated transcripts thought to regulate OFV genome replication (Kondo et al., 2014) (Table S3). Together, these results suggest that the reads were mainly derived from the poly(A)^+^ RNAs transcribed from the (-)genome RNA of OFV, thus further supporting the possibility that the OFV gene expression occurs in mites as well as in plants.

The transcriptional gradient from 3′ (*N*) to 5′ (*L*) (3′–5′ polar gradient of mRNA production) is commonly observed for non-segmented rhabdoviruses (Dietzgen et al., 2017; Horie et al., 2021). However, the density of reads mapped to each OFV gene-coding and trailer/leader region, calculated based on the number of reads per 100 nucleotides, did not suggest a transcriptional gradient in the OFV RNA1 segment in either the plant and the mite datasets. Nevertheless, the *G* gene, located at the 5′-proximal region, showed the lowest read density compared to other RNA1-encoded genes (Fig. 3D and Table S3). It should also be noted that the relative proportion of the reads mapped to RNA2 (*L* mRNA) was much lower in the leaf dataset (∼1/8) than in the mite dataset (∼1/2) (Fig. 3D), suggesting that the ratio of OFV RNA2 to RNA1 accumulation or gene expression level may be higher in mites than in plants.

### 3.5 Analysis of OFV small RNA accumulation in plants and viruliferous mites

**Fig. 4:**
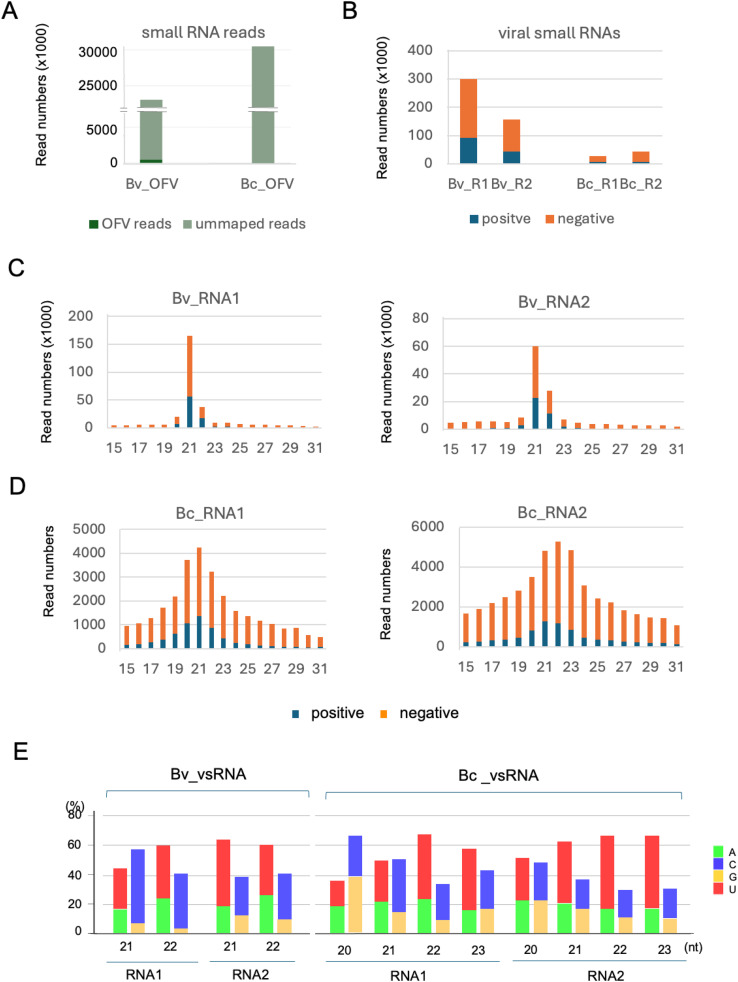
**OFV-derived small RNA (vsRNA) profiles in host plant and mite vector. A,** total amount of vsRNA reads derived from deep sequence analysis (**A**). **B**, proportion of minus (-) and plus (+)-strand vsRNAs (15–31 nt) in the host plant (OFV-infected *B. vulgaris* var*. cicla* leaf, Bv_OFV) and viruliferous *B. californicus* s.l. (Bc_OFV) samples. **C** and **D**, size distribution of vsRNA reads (15–31 nt) per million total small RNAs in *B. vulgaris* var. *cicla* (**C**) and *B. californicus* s.l. (**D**) samples. **E**, proportions of 5ꞌ-terminal nucleotides of vsRNAs in the host plant and the mite vector. The bar graphs indicate the percentage of the 5ꞌ-terminal nucleotides of each vsRNA size (21 and 22 for plants and 20–23 nt for mites).

RNA silencing is a small-RNA-mediated down-regulation mechanism of cellular gene expression (Hannon, 2002). In plants, insects and other eukaryotes, RNA silencing operates as an innate antiviral defense that limits virus accumulation or spread through the generation of viral-derived small RNAs that guide the Argonaute-protein-containing effector complex for sequence-specific RNA turnover (Ding and Voinnet, 2007). The host Dicer or Dicer-like (DCL) protein recognizes and cleaves double-stranded RNAs (dsRNAs) generated during RNA virus replication (dsRNA replicative form) to produce viral-derived small interfering RNAs (siRNAs) (Ding and Voinnet, 2007). Thus, investigating the presence of viral-derived small RNAs (vsRNAs) in the cells could provide an indicative observation suggesting the occurrence of virus replication, as is the case for some plant RNA viruses (de Haro et al., 2017; Yang et al., 2018). To this end, small RNA fractions from OFV_Cym07-infected Swiss chard leaves and viruliferous *B. californicus* s.l. (Cym07Bc_JP) were subjected to deep sequence analysis to investigate the accumulation of vsRNA. A total of 23.1 M and 26.5 M raw reads ranging from 15 to 31 nt in length were obtained from the plant and mite samples, respectively (Fig. 4A). The size distribution of small RNA reads from the leaf sample showed a prominent range of 21−24-nt (Fig. S5), a pattern commonly observed in plant small RNAs (Yang et al., 2018). In contrast, the small RNAs from the mite sample displayed a peak at 22 nt (Fig. S5), similar to the size distribution profile of dust mites, although in that case, the peak was at 24 nt (Mondal et al., 2018).

Around 2.0 % (0.46 M) of small RNA reads derived from the Swiss chard sample were mapped to OFV_Cym07 genome sequences, while only 0.2 % (0.07 M) of small RNA reads derived from the mite sample were mapped to viral genome sequences (Fig. 4B). Among mapped small RNAs, a higher proportion of vsRNAs in the leaf data set were mapped to RNA1 (65.8 %). In the mite data set, a higher proportion of vsRNAs were mapped to RNA2 (60.9 %) (Fig. S6A). These results were in line with our RNA-seq analysis result suggesting a higher ratio of OFV RNA2 to RNA1 accumulation or gene expression in mites than in plants (Fig. 4D).

In both plant and mite datasets, much higher proportions of vsRNA were derived from the virus-negative strand genome (RNA1/RNA2; 69.0 %/71.3 % in plant and 78.1 %/82.8 % in mites) (Figs. 4B and S6B). In the plant dataset, vsRNAs sizes were predominantly 21 and 22 nt, with the former being the main size (Fig. 4C). On the other hand, vsRNAs in the mite data set showed a rather broad size distribution, with prominent sizes of 20–22 nt for RNA1 and 21–23 nt for RNA2 (Fig. 4D). As for the 5′-terminal nucleotides of vsRNAs, which usually show an adenine (A)/uracil (U) bias in plants and insects (Li et al., 2017; Xu et al., 2012), a clear A/U bias was observed for RNA2-derived vsRNAs but not for RNA1-derived vsRNAs in both datasets (Fig.4E).

**Fig. 5:**
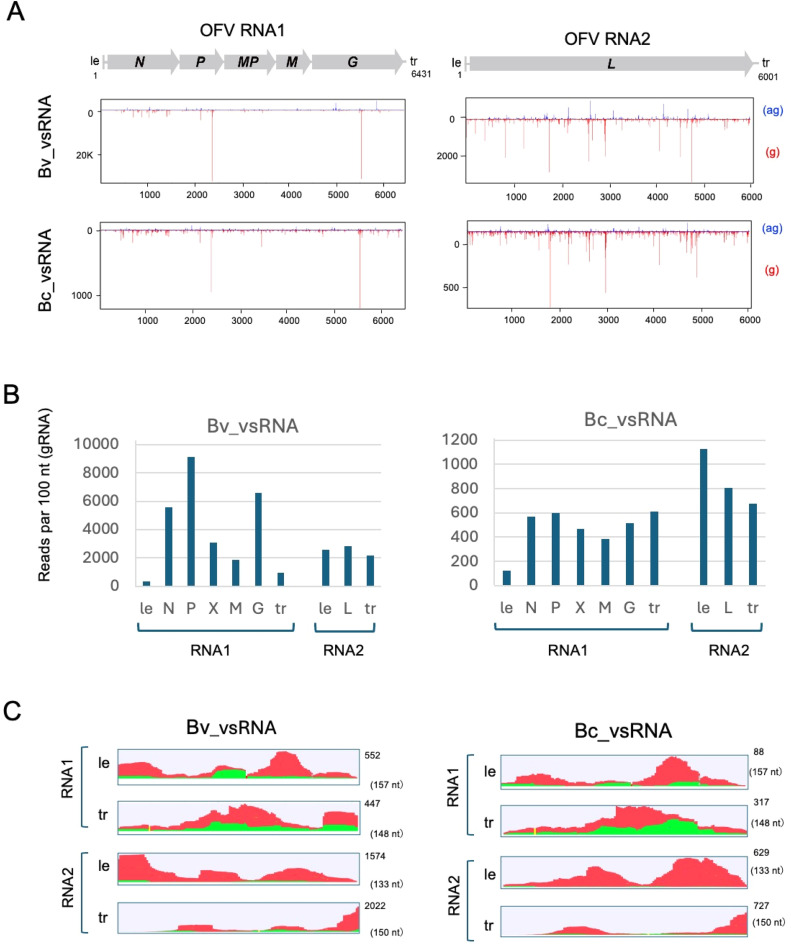
**Distribution of OFV-derived small RNA (vsRNA) in the virus genome. A,** Distribution of vsRNA along the genome sequences of OFV_Cym07. Blue and red color coding indicate the reads mapped to the antigenome (ag) and the genome (g) strands, respectively. **B,** the vsRNA reads from *B. vulgaris* var. *cicla* (left side) and *B. californicus* s.l. (right side) samples mapped to each gene of the OFV segment (vertical axis shows number of reads per 100 bases). **C,** read depth coverage of the vsRNAs on the leader (*le*) and trailer (*tr*) regions of OFV_Cym07 in *B. vulgaris* var. *cicla* (left side) and *B. californicus* s.l. (right side) datasets. Green and red color coding indicate the antigenome strand (positive) and genome strand (negative), respectively.

Mapping of plant and mite vsRNAs to the OFV genome showed that vsRNAs were distributed throughout the OFV genome, including in the non-transcribed regions, with several prominent vsRNA hotspots located at the similar genomic positions between the plant and mite data (Fig. 5A). Next, the density of vsRNAs mapped to each OFV gene-coding and trailer/leader region were calculated based on the number of vsRNA per 100 bases. The result showed that the density of vsRNAs varied widely among regions (Fig. 5B). In the plant, vsRNA density was high in the *P* and *G* gene coding regions, likely due to in part the presence of prominent vsRNA hotspots in these regions (Fig. 5A). The density vsRNA was relatively lower in the trailer/leader regions of RNA1 but higher in those of RNA2 (Fig. 5B). Density of vsRNA in the mites was comparable across the OFV gene-coding and trailer regions of RNA1 and RNA2. At the same time, it was relatively low in the leader region of RNA1 but notably high in the leader region of RNA2 (Fig. 5B). The distribution of vsRNAs in the leader and trailer regions of RNA1 and RNA2 showed a relatively similar pattern between plant and mite data, except for the leader sequence of RNA2, which appeared to show a different distribution profile (Fig. 5C).

## 4 Discussion

Arthropods (specifically insects and mites) can be vectors of many pathogenic viruses that affect plants, humans, and animals and studying vector–virus interactions is therefore important for understanding virus spread, epidemiology and their control. The mode of virus transmission involving arthropod vectors has only been studied in depth for certain groups, such as mosquitoes, ticks, and phytophagous insects, whereas information on mite–plant–virus interactions is largely unknown (Hogenhout et al., 2008; Whitfield et al., 2015). To gain insight into the replication potential of plant rhabdoviruses persistently transmitted by phytophagous mites, we utilized RT-qPCR, western blotting and next-generation sequencing technology to analyze viruliferous *B. californicus* s.l. mites (Cym07Bc_JP) (Fig. 1) carrying a bisegmented dichorhavirus (OFV orchid strain, Cym07). Our analyses indicated the increasing virus titer after virus acquisition (Figs. 2D, E and S3C) and accumulation of viral mRNA and vsRNA in the mites. The expression and accumulation patterns of these OFV-derived RNA molecules in the mites appeared to be different from those observed in plants (Figs. 3, Fig. 4, Fig. 5), while some portions of these RNA molecules were potentially acquired during feeding on infected plants (see Fig S4 and data not shown) and therefore the viral RNA profile in mites needs to be verified with more samples. These data indicate a replicative mode of transmission by the mite vectors, and also imply the existence of an antiviral RNA silencing mechanism in phytophagous mites.

The results of our RNA-seq analysis suggested that the expression of OFV mRNA also occurs in *B. californicus* s.l. mites, similarly to its expression in the plants (*T. expansa*), and thus the transcriptional attenuation known to occur in non-segmented rhabdoviruses (Dietzgen et al., 2017) did not seem to be a major factor affecting RNA1 gene transcriptions in either host (Fig. 3). The RNA-seq results also suggested that the ratio of OFV RNA2 accumulation or gene expression levels relative to those of RNA1 may be higher in mites than in plants (Fig. 3 and see below). The transcription of leader or trailer regions (genomic or antigenomic strand, respectively) as non-coding polyadenylated RNAs has also been confirmed in a classical plant nucleorhabdovirus (sonchus yellow net virus). However, this has not been observed in cytoplasmically replicating animal rhabdoviruses, which lack a poly-A tail (Kondo et al., 2014; Wagner et al., 1996; Wagner and Jackson, 1997). OFV and nucleorhabdoviruses are thought to replicate in the nucleus, and the leader RNAs may be involved in the regulation of genome replication and transcription in the nucleus (Kitajima et al., 2001; Kondo et al., 2013; Martins et al., 1998). In this study, the observation that the (+)leader RNA of OFV RNA2 is transcribed at low levels in both plants and mites may be related to the expression level of the *L* mRNA in RNA2, which is much lower than that of RNA1-encoded genes. This relation pattern is particularly pronounced in plants, even though RNA1 and RNA2 accumulate to the same extent (Fig. 3). It is therefore possible that, unlike the regulation of gene expression in non-segmented rhabdoviruses, segmented rhabdoviruses may have evolved a specific gene expression mechanism for *L* mRNA in the second segment.

Our deep sequence analysis confirmed the accumulation of OFV vsRNAs in plants (Swiss chard) and *B. californicus* s.l. mites with the majority of vsRNAs derived from minus-stranded genome RNA, and this trend was more pronounced in mites than in plants (Fig. 4). In OFV-infected plants, a predominant accumulation of 21–22 nt vsRNAs was observed, which is attributed to two Dicer-like (DCL) proteins, DCL2 and DCL4 (Deleris et al., 2006). In contrast, the OFV vsRNAs from mites showed a broad size distribution predominated by 20–23-nt vsRNAs being particularly prominent (Fig. 4). The antiviral RNA-silencing mechanisms and their key factors in phytophagous mites remain to be explored. Antiviral silencing against arboviruses (e.g., flaviviruses) in ticks has been studied, showing a predominance of 22-nt vsRNAs (Schnettler et al., 2014). In the case of (-)RNA viruses such as rhabdoviruses, there is little accumulation of naked dsRNA replication intermediates in the cells (Dietzgen et al., 2017; Weber et al., 2006). Replication of OFV and nucleorhabdovirus occurs in the nuclear regions. Although antiviral silencing, particularly that involving DCL4, contributes to the defense against cytoplasmic-replicating RNA viruses (Blevins et al., 2006), DCL4 has also been thought to be active in the nucleus for the small RNA production (Pontes et al., 2013). OFV and nucleorhabdovirus genomic RNAs are therefore likely to be targeted by antiviral silencing, resulting in high levels of negative-stranded vsRNA in the infected cells (Fig. 5). However, it is still unknown how these negative-stranded vsRNAs are generated by the RNA-silencing machinery, since their genomic RNAs are mostly covered by nucleocapsid protein. In the context of viral counter-defense, the OFV *ORF3* gene, which encodes a putative MP, has been suggested to suppress systemic RNA silencing in *N. benthamiana* (Leastro et al., 2022). Whether the *ORF3*-encoded protein functions similarly in other plant hosts and mites, as well as whether its function involves other viral factor(s), are interesting questions. Our data provide insight into antiviral RNA-silencing responses in phytophagous mites, and other antiviral mechanisms in mites also warrant further investigation.

The OFV G protein appears to have typical glycoprotein characteristics with putative signal and transmembrane domains, but it remains unclear whether the OFV G protein has a function in virus entry into vector cells similar to that of rhabdovirus G proteins (Kondo et al., 2009). Unlike the classical rhabdoviruses, which have an envelope with G protein spikes in their mature virions, OFV and other dichorhaviruses appear to lack this envelope (Dietzgen et al., 2017; Kondo et al., 2009). Instead, in plant cells, dichorhavirus particles are likely to associate with host nuclear membranes, forming a unique structure known as a spoke wheel (SW) and accumulating in the perinuclear space (Dietzgen et al., 2017; Kondo et al., 2003). Since another dichorhavirus (coffee ring spot virus) G protein localizes to the nuclear envelope in the plant cells (Ramalho et al., 2014), it is possible that the membrane-associated SW structures are formed through the involvement of the G protein. Similar membrane-associated SW structures have been observed in mites harboring a dichorhavirus (Kitajima and Alberti, 2014), suggesting that the structure may contribute to virus transmission via mite vector, similar to G protein-spiked rhabdovirus virions. The *G* gene is the most diverse gene in the genome of plant rhabdoviruses and the region of potential positive selection, as in other animal rhabdoviruses (Holmes et al., 2002). In fact, long-term transmission of OFV orchid strain to Swiss chard plants via mite vectors likely resulted in an accumulation of non-synonymous substitutions in the *G* gene as compared to other genomic regions (Fig. 2). In contrast, repeated mechanical inoculation of the OFV orchid strain (So isolate) into *T. expansa* leaves and long-term maintenance (over one year) did not result in a deletion or frequent substitutions in the *G* gene (HK unpublished results). These differences may be related to the fitness of OFV in the specific host plants rather than the compatibility with the vector mite for transmission.

Most members of the genus *Dichorhavirus*, which is characterized by a bisegmented genome, are primarily distributed in Central and South America (Dietzgen et al., 2018). However, the orchid strain of OFV is unique within the genus in terms of its worldwide distribution. Furthermore, this orchid strain has been reported to cause disease in citrus plants outside of the American continent (in Hawaii and South Africa) (Cook et al., 2019; Olmedo-Velarde et al., 2021). The worldwide distribution of OFV orchid strain has likely been facilitated by international trade or germplasm exchange in orchids (Peng et al., 2013). Besides, OFV—at least the orchid strain, but not for other dichorhaviruses and cileviruses—could be systemically infectious in their natural hosts such as *Cymbidium* spp. and some other orchid species (Fig. S1) (Blanchfield et al., 2001; Dietzgen, et al., 2017; Freitas-Astua et al., 2018; Kondo et al., 2003). Furthermore, as suggested by this and previous studies, the vector *B. californicus* s.l. transmits OFV in a replicative manner and was found in relatively cooler areas, whereas the vector *B. yothersi* transmits cileviruses in a non-replicative manner and prefers warmer geographical areas (Freitas-Astua et al., 2018; Kondo et al., 2003; Roy et al., 2015a). From an epidemiological perspective, these characteristics may be determining factors in the spread of this virus in wider areas outside of the American continent.

## 5 Conclusion

In this study, we present molecular evidence for the increasing virus titer following virus acquisition and gene expression and small RNA accumulation of bisegmented rhabdoviruses in mite vectors, indicating the occurrence of virus replication in the mite. Many classical plant rhabdoviruses are persistently transmitted by insects in a replicative manner, and their infection mechanisms in plant hosts and insect vectors have been studied in detail, mainly because infectious viral cDNA clones have been established for some non-segmented plant rhabdoviruses (classical nucleo- and cytorhabdoviruses) (Gao et al., 2023; 2024; German et al., 2020; Jackson et al., 2018; Liang et al., 2023; Xavier et al., 2024). However, infectious viral cDNA clones have not been established for bisegmented plant rhabdoviruses. Further detailed studies on the replication of bisegmented plant rhabdoviruses in plant and mite vectors will lead to a better understanding of vector specificity and co-evolution of rhabdoviruses with theses vectors.

## Funding

This research was supported by grants through the Grants-in-Aids for Scientific Research (KAKENHI 23K26907; 23K18029; 21H05035; 21K18222) from the JSPS, the Ministry of Education, Culture, Sports, Science, and Technology (MEXT) of Japan, as well by the Ohara Foundation for Agriculture Research, Kurashiki, Japan.

## CRediT authorship contribution statement

**Hideki Kondo:** Writing – review & editing, Writing – original draft, Visualization, Software, Investigation, Funding acquisition, Data curation, Conceptualization. **Miki Fujita:** Investigation. **Paul Telengech:** Investigation. **Kazuyuki Maruyam:** Investigation. **Kiwamu Hyodo:** Writing – review & editing, Validation. **Aline Daniele Tassi:** Writing – review & editing, Formal analysis, Data curation. **Ronald Ochoa:** Writing – review & editing, Formal analysis, Data curation. **Ida Bagus Andika:** Writing – review & editing, Writing – original draft. **Nobuhiro Suzuki:** Writing – review & editing, Funding acquisition.

## Declaration of competing interest

The authors declare that they have no known competing financial interests or personal relationships that could have appeared to influence the work reported in this paper. Mention of trade names or commercial products in this publication is solely for providing specific information and does not imply recommendation or endorsement by the USDA; USDA is an equal opportunity provider and employer.

## Appendix Supplementary materials

Image, application 1
