# Supplementary material for: Evidence for the replication of a plant rhabdovirus in its arthropod mite vector
Source: Virus Res. 2025 Jan 4;351:199522. doi: 10.1016/j.virusres.2024.199522 (PMC11757783; doi:10.1016/j.virusres.2024.199522)

**Supplementary material**

**
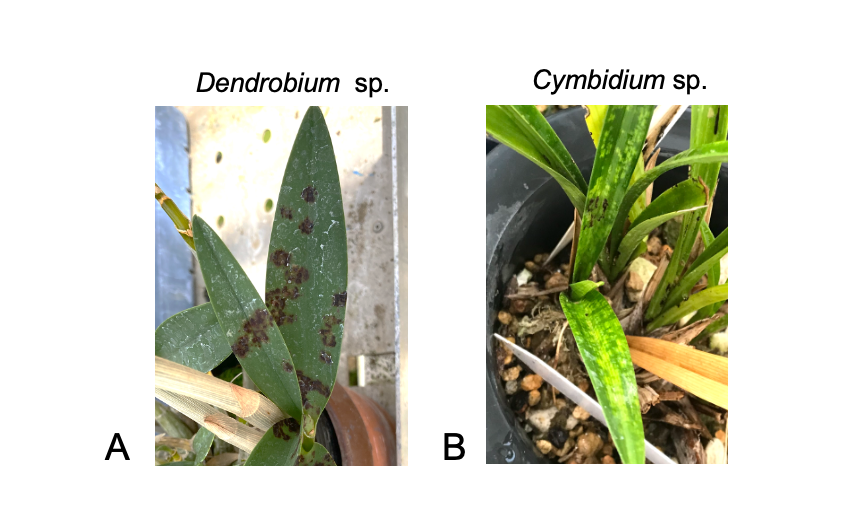
**

**Fig. S1. Symptoms of OFV_Cym07 in plant hosts inoculated with viruliferous *B. californicus* s.l. A and B**, symptom in the OFV-infected leaves of *Dendrobium* sp**. (A)** and uninoculated systemic leaves of *Cymbidium* sp. **(B).** Photos were taken in the spring of the following year after inoculation feeding*.*

**
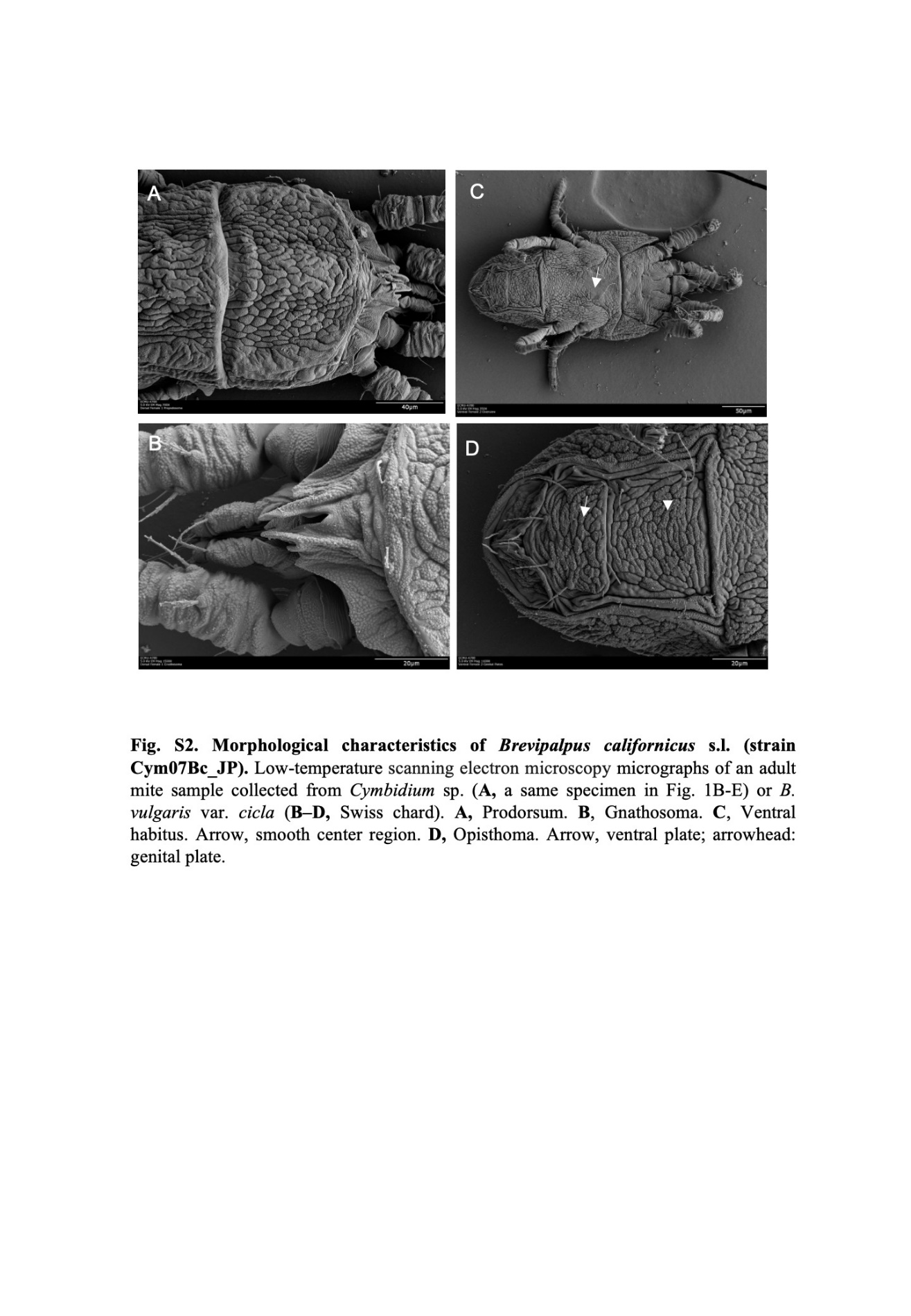
**

**Fig. S2. Morphological characteristics of *Brevipalpus* *californicus* s.l. (strain Cym07Bc_JP).** Low-temperature scanning electron microscopy micrographs of an adult mite sample collected from *Cymbidium* sp. (**A**, the same specimen in Fig. 1B–E) or *B. vulgaris* var. *cicla* (Swiss chard, **B–D)**. **A**, prodorsum. **B,** gnathosoma. **C**, ventral habitus. Arrow, smooth center region. **D,** opisthoma. Arrow, ventral plate; arrowhead: genital plate.

**
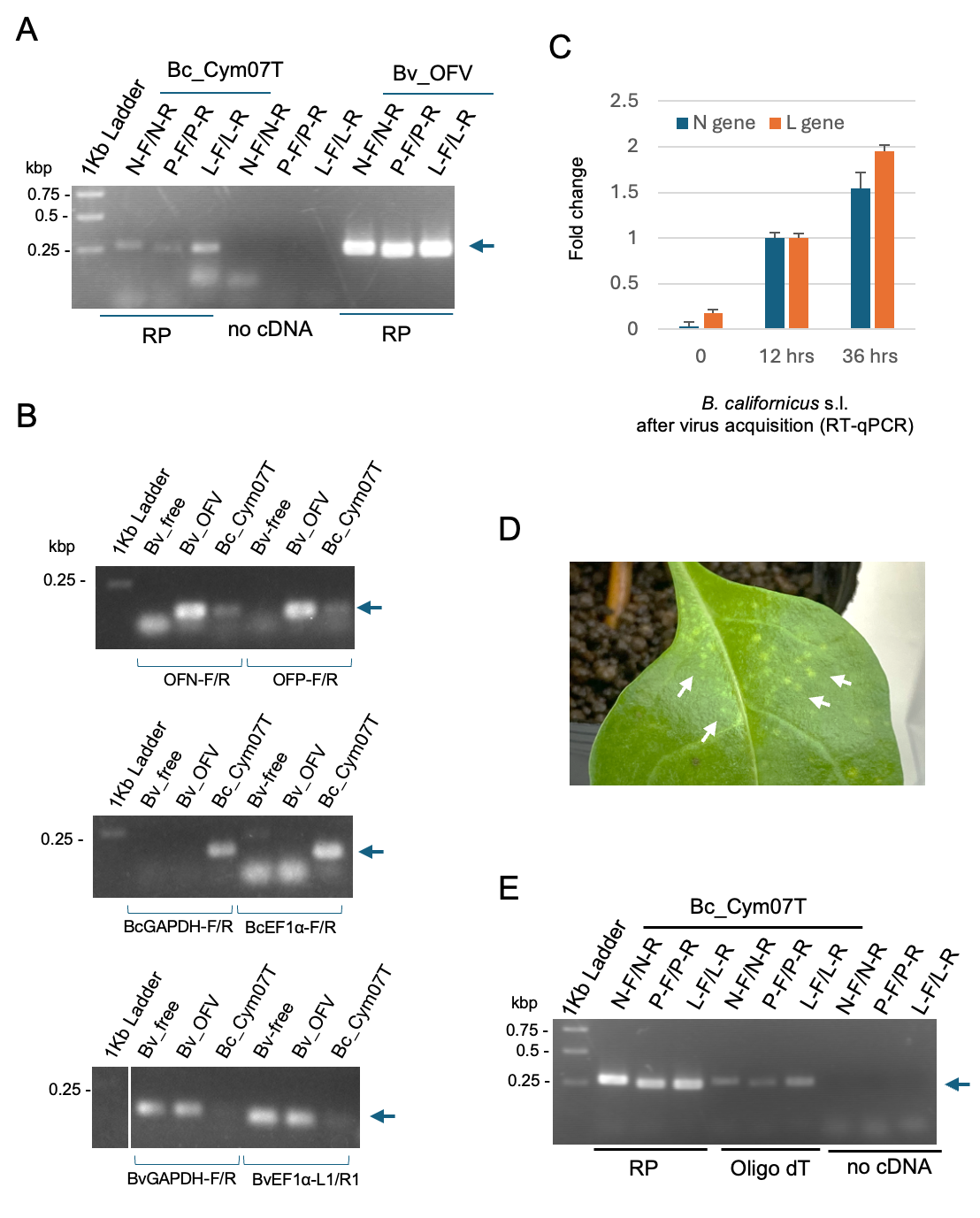
**

**Fig. S3. Detection of OFV_Cym07 in plant and mite samples. A, B** and **E,** RT-PCR of plant and mite samples (Bv_OFV and Bc_Cym07T) using random primers (RT) or oligo dT primers for the cDNA synthesis. PCR amplification was performed for 30 (**A** and **B**) or for 35 (**E**) temperature cycles. 1Kb Ladder: Molecular weight marker. The gels were stained with ethidium bromide. The PCR reactions without cDNA (no cDNA) were used as a negative control. All primers for OFV genes (*N, P* and *L* genes) and plant/mite genes (*Bc/BvGAPDH* and *Bc/BvEF1a*) are listed in Table S1. **C,** OFV accumulation in the adult mites at different time points after virus acquisition (12 hours, and 36 hours reared on virus-free leaves for a further 24 hours) measured by RT-qPCR (*N* and *L* genes). Data are presented as the fold change of viral accumulation (the value at 12 hours was set to 1) with three technical replicates. **D,** A symptom in a leaf of *B. vulgaris* var. *cicla* infested with *B. californicus* s.l. mites (10 adults for each leaf) after 5 days after virus acquisition. The photograph was taken 18 days after inoculation feeding.


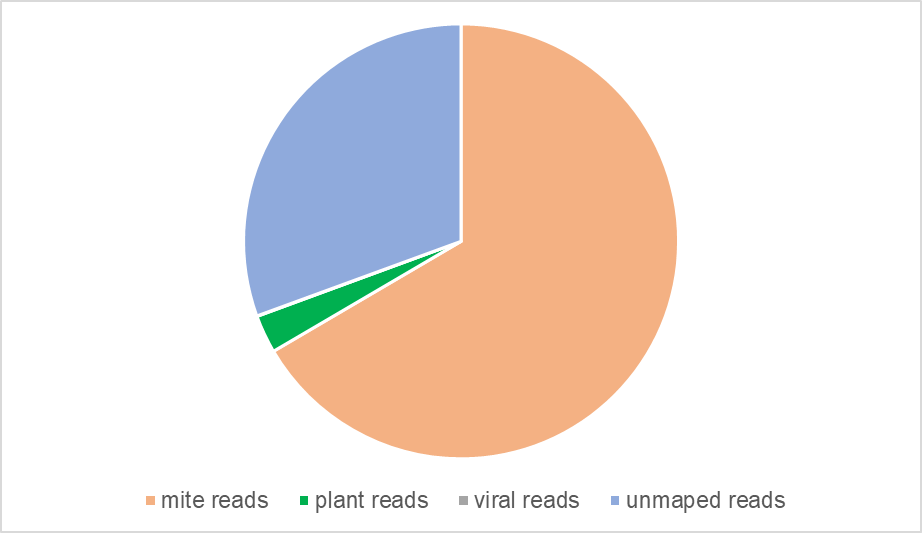


**2.83%**

**0.03%**

**66.57%**

**30.57%**

(48.3 M raw pair-end reads)

Viruliferous *B. californicus* s.l. (Cym07Bc_JP)

**Fig. S4. Number of raw reads in the viruliferous *B. californicus* s.l. (Cym07Bc_JP) library mapped to OFV, mite and plant genome sequences.** The charts show the percentage of raw reads, estimated using a read mapping approach with the genomic sequences of *Brevipalpus yothersi* (GCA_003956705 including an insect Cardinium endosymbiont (*Bemisia tabaci*, NZ_ML143135.1) as an alternative endosymbiotic bacterium, *Beta vulgaris* subsp. *vulgaris* (sugar beet) (GCF_026745355, genome assembly EL10.2) or OFV.

**
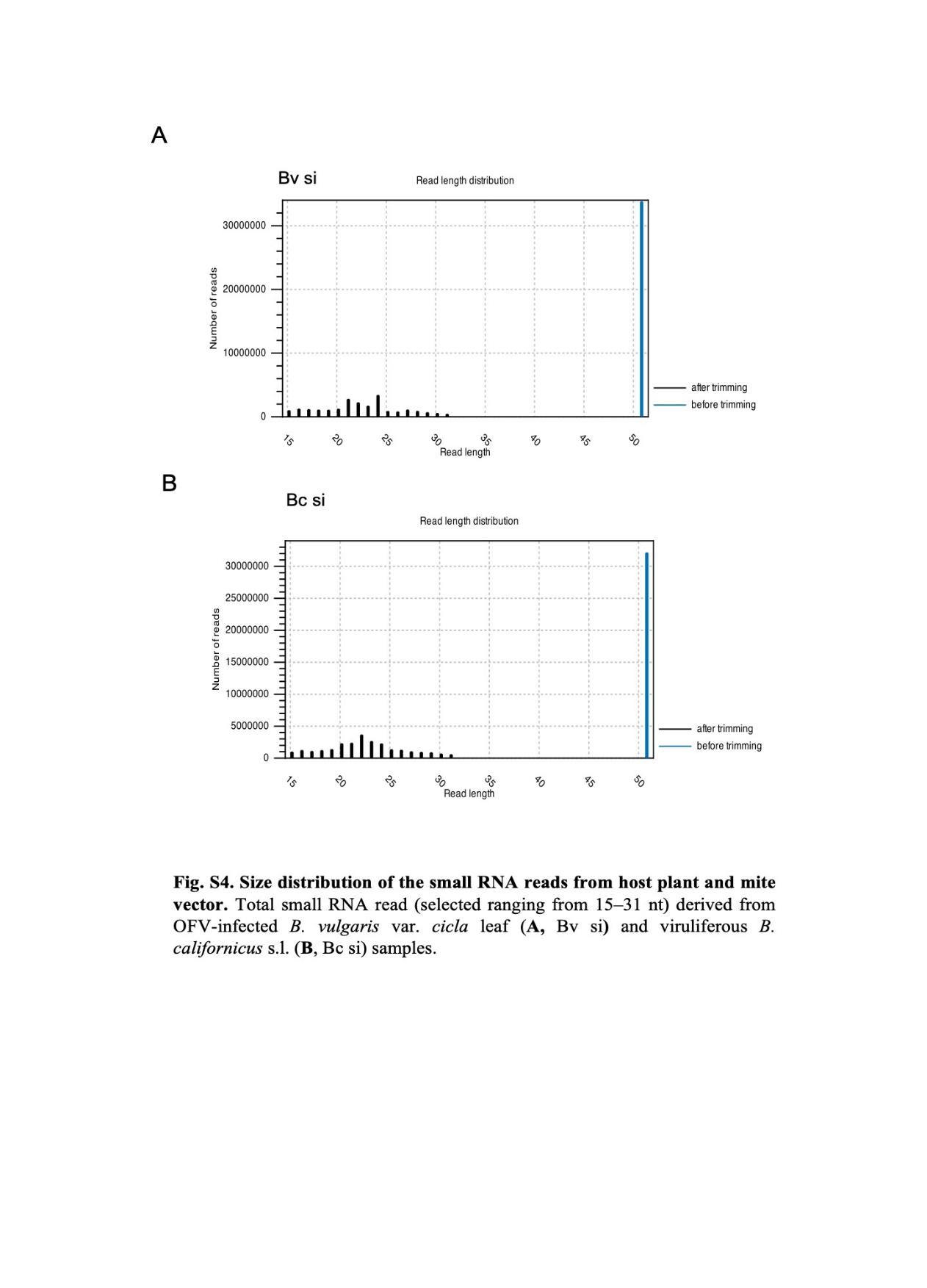
**

**Fig. S5. Size distribution of the small RNA reads from host plant and mite vector.** Total small RNA read (selected range from 15–31 nt) derived from OFV-infected *B. vulgaris* var. *cicla* leaf (**A**, Bv si) and viruliferous *B. californicus* s.l. (**B**, Bc si) samples.


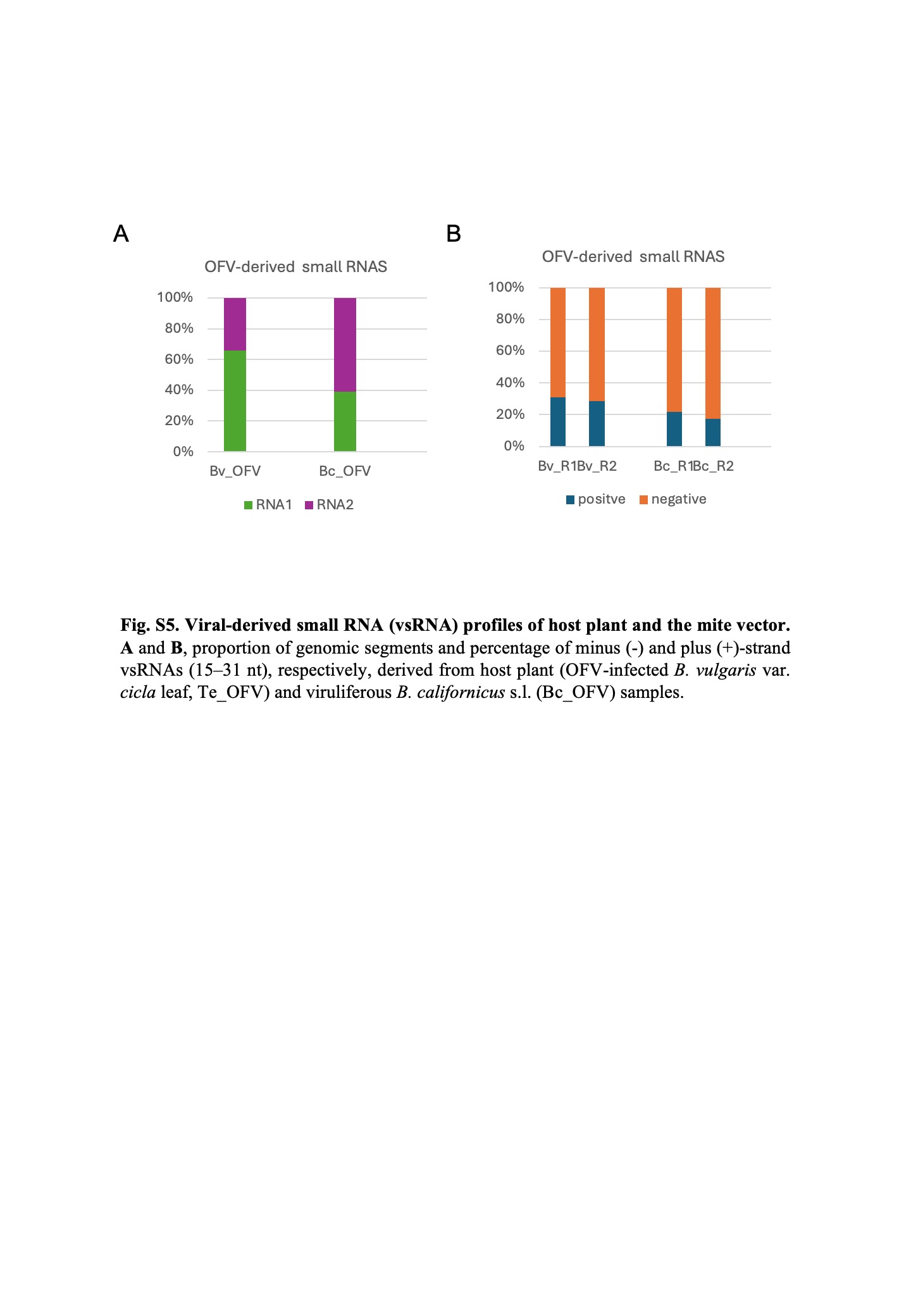


**Fig. S6. Polarity profile of OFV-derived small RNA (vsRNA) in the host plant and the mite vector. A** and **B,** proportion of minus (-) and plus (+)-strand vsRNAs (15–31 nt) in the host plant (OFV-infected *B. vulgaris* var. *cicla* leaf, Te_OFV) and viruliferous *B. californicus* s.l. (Bc_OFV) samples.

**Table 1.** Primer list for the detection of three OFV genes, plant or mite reference genes.

Name sequence (5’-3’) amplicon

Primers for RT-PCR

N-F GTGTGTCACTCTAATAGCCAGGATGGC  273 bp

N-R GGGTCTGGCGGATGGTGGTGTGAACAG

P-F GAGAAAACATCCGGATTCACTAGACAC   254 bp

P-R CGGATAAGCTGGCCTGATGTTTCAGGG

L-F AGATGAGGACGATTTTCTGGAGGAACG  253 pb

L-R GCTCCGATAGGCTTGGGAGTTACTCTG

Primers for RT-PCR or RT-qPCR

OFN-F AGGCATACAATGGCCTGTCC 100 bp

OFN-R AACAGACCTCTGCGAGTTGG

OFP-F GAAGCTCCTCGTGCTCAACT 92 bp

OFP-R ACGCGGGGTTATCCGTTATC

OFL-F AGCCCCACCAGGGTACTAAT 106 bp

OFL-R TGGCCTGAAAATCTCCCCAC

BvGAPDH-F^*^ GCTTTGAACGACCACTTCGC not provided

BvGAPDH-R^*^ ACGCCGAGAGCAACTTGAAC

BvEF1α-L1^**^ GATTCCCACCAAGCCTATGG not provided

BvEF1α-R1^**^ GATGACACCAACAGCGACAG

BcGAPDH-F^***^ TTGTCAAGCGATCAAAGCTGC 148 bp

BcGAPDH-R^***^ TCGTTTGCTCAGCTGGATACC

BcEF1α-F^***^ GTGCTCGATTGTCACACTGC 129 bp

BcEF1α-R^***^ CACAATGGCAGCATCACCAG

* Chiurugwi, T., Holmes, H. F., Qi, A., Chia, T. Y., Hedden, P., Mutasa-Göttgens, E. S. (2013). Development of new quantitative physiological and molecular breeding parameters based on the sugar-beet vernalization intensity model. The *Journal of Agricultural Science* 151(4), 492-505.

**Mutasa-Göttgens, E. S., Joshi, A., Holmes, H. F., Hedden, P., Göttgens, B. (2012). A new RNASeq-based reference transcriptome for sugar beet and its application in transcriptome-scale analysis of vernalization and gibberellin responses. *BMC genomics*, 13, 1-18.

*** Primers for the *B. californicus* GAPDH and EF1α genes’ candidates were designed based on the RNA-seq sequence contigs obtained from the BLAST search. These *B. californicus* sequences were compared with the corresponding genomic sequences of *B. yothersi* isolate EMBRAPA2013 (scaffold219 and scaffold457) and showed 92% and 95% nucleotide identity, respectively. A region containing the predicted intron of each gene was selected for PCR (data not shown).

**Table S2.** Comparison of viral sequences obtained by RNA-Seq, with the reference virus sequence deposited in GenBank (Acc. No LC222629 and LC222630).

**
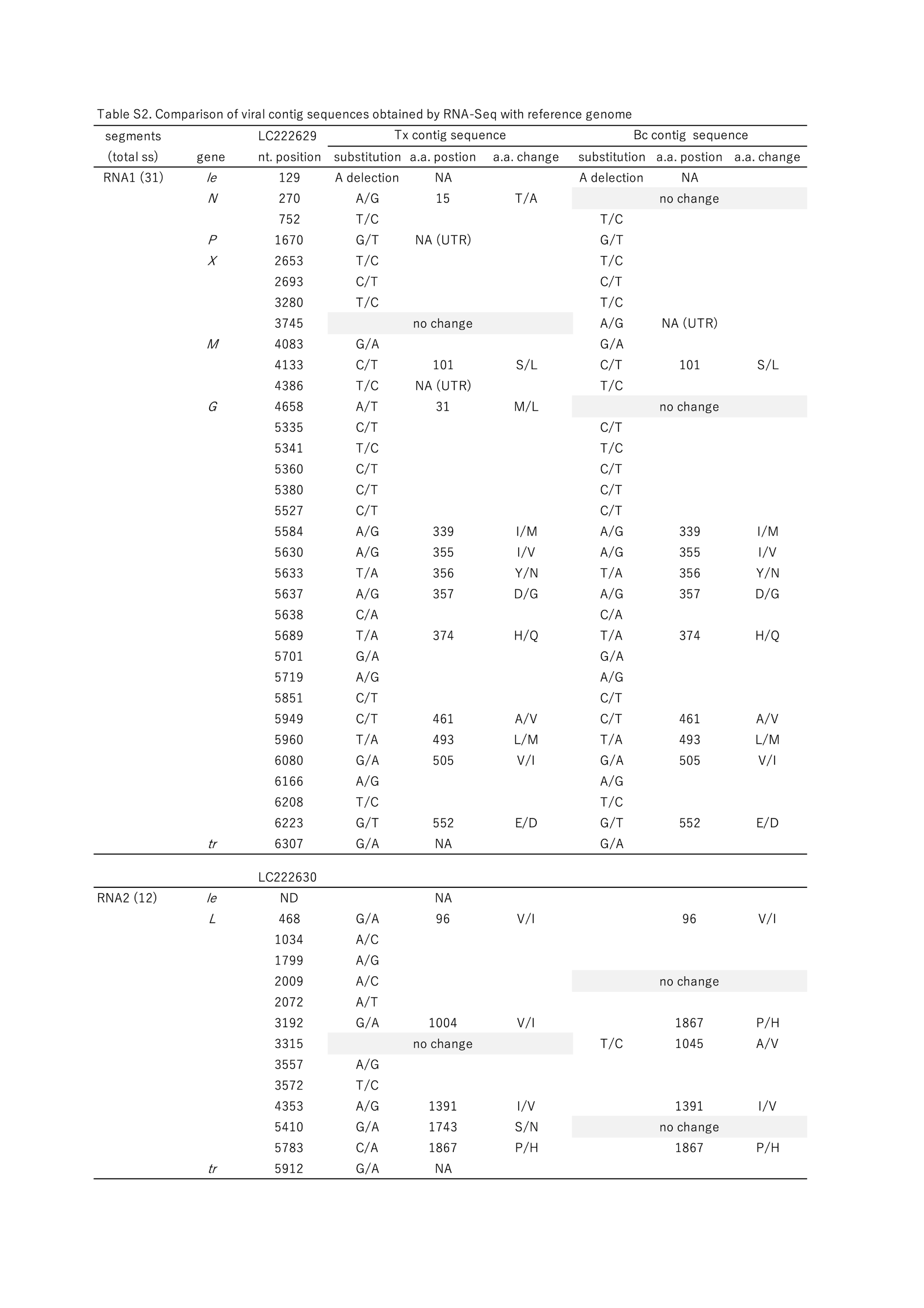
**

**Table S3.** The number of reads mapped to each OFV gene region (mRNA).


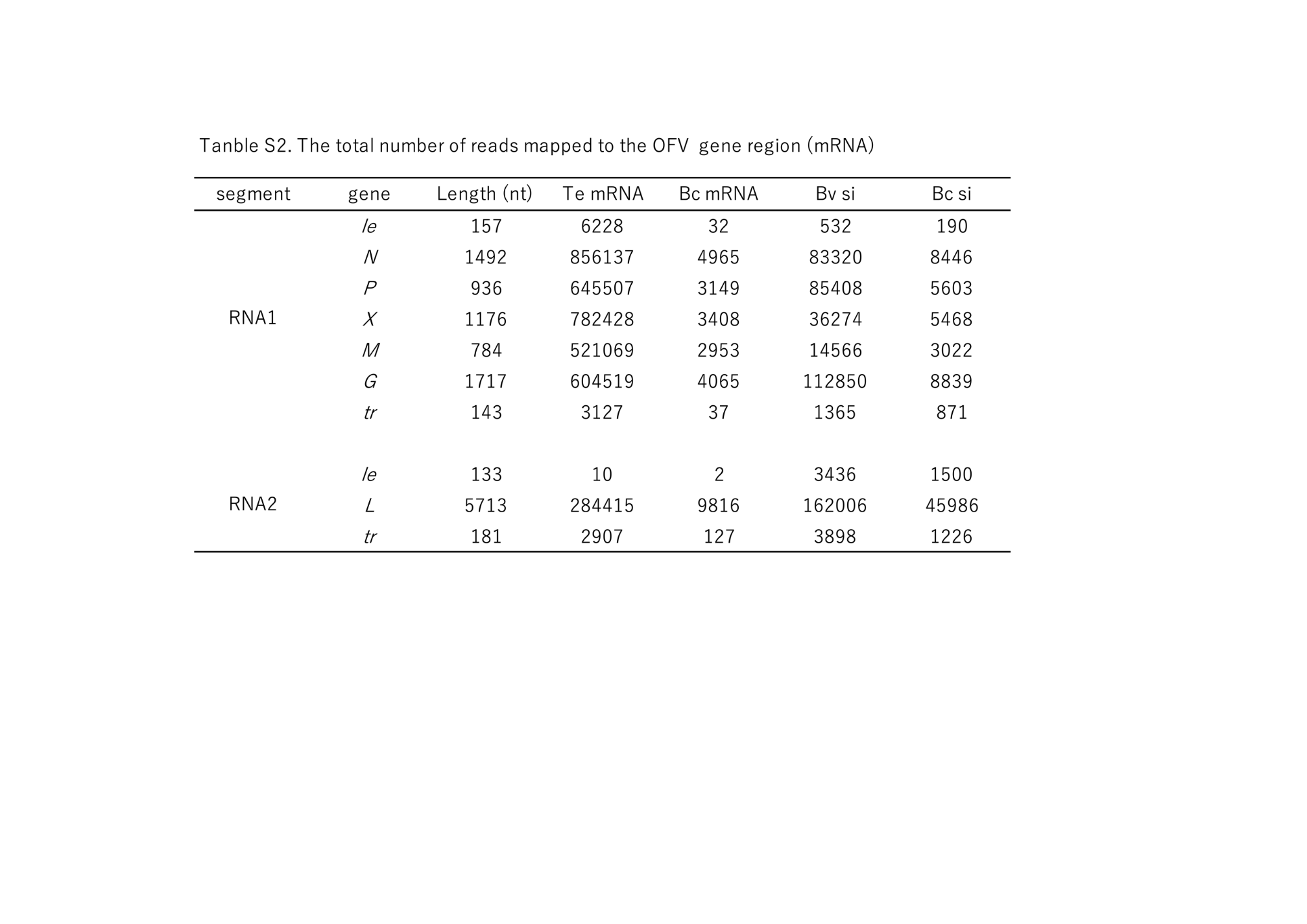

Supplement: Supplementary file 1 [file mmc1.docx]
